# NEIGHBORHOOD DISADVANTAGE, IMAGING BIOMARKERS OF ALZHEIMER’S DISEASE, AND COGNITION IN DIVERSE OLDER ADULTS

**DOI:** 10.1093/geroni/igad104.3767

**Published:** 2023-12-21

**Authors:** Erica Fan, Sarah Royse, Beth Snitz, Tharick Pascoal, C Shaaban, Brian Lopresti, Victor Villemagne, Ann Cohen

**Affiliations:** University of Pittsburgh, School of Public Health, Pittsburgh, Pennsylvania, United States; University of Pittsburgh, Pittsburgh, Pennsylvania, United States; University of Pittsburgh, Pittsburgh, Pennsylvania, United States; University of Pittsburgh, Pittsburgh, Pennsylvania, United States; University of Pittsburgh, Pittsburgh, Pennsylvania, United States; University of Pittsburgh, Pittsburgh, Pennsylvania, United States; University of Pittsburgh, Pittsburgh, Pennsylvania, United States; University of Pittsburgh, Pittsburgh, Pennsylvania, United States

## Abstract

Neuroimaging biomarkers of Alzheimer’s disease (AD) are well-validated in White populations. However, it remains unclear how these biomarkers are associated with cognition in non-White populations disproportionately affected by systemic socioeconomic discrimination. We investigated whether neighborhood disadvantage modifies associations of neuroimaging biomarkers with cognition in a diverse cohort of 198 adults ≥50 years, 50% of whom are Black. Outcomes included cognitive impairment and neuropsychological test scores. Predictors were indices of neurodegeneration, vascular damage, and brain amyloid burden, quantified using MR-based measures of cortical thickness and white matter hyperintensities (WMH) and [11C]PiB PET, respectively. We measured neighborhood disadvantage using area deprivation index (ADI), a composite measurement of socioeconomic conditions in a census tract. In linear models, higher ADI, neurodegeneration, and WMH were separately associated with worse cognition. We found an ADI*neurodegeneration interaction on delayed recall memory (p=0.08). Neurodegeneration-positive participants had worse story delayed recall compared to neurodegeneration-negative regardless of ADI (β=-1.57, p=0.01). In neurodegeneration-negative participants, higher ADI is associated with worse delayed recall (β=-0.04, p=0.004): those with a high ADI exhibited low delayed recall, comparable to neurodegeneration-positive participants. ADI was not associated with cognition in neurodegeneration-positive participants. WMH and ADI interacted in models of impairment, visual memory, and visual copy (interaction p’s< 0.10). In those with high ADI, greater WMH was associated with worse cognition (β=-0.30, p=0.02) while participants with low ADI scored well regardless of WMH volume. Taken together, the relationship between neuroimaging biomarkers and cognition differs by neighborhood socioeconomic conditions, highlighting the need for clinical strategies that acknowledge structural disadvantage.

